# Uridine as a Regulator of Functional and Ultrastructural Changes in the Brain of Rats in a Model of 6-OHDA-Induced Parkinson’s Disease

**DOI:** 10.3390/ijms241814304

**Published:** 2023-09-19

**Authors:** Nina I. Uspalenko, Alexei A. Mosentsov, Natalia V. Khmil, Lyubov L. Pavlik, Natalia V. Belosludtseva, Natalia V. Khunderyakova, Maria I. Shigaeva, Vasilisa P. Medvedeva, Anton E. Malkov, Valentina F. Kitchigina, Galina D. Mironova

**Affiliations:** 1 Institute of Theoretical and Experimental Biophysics, Russian Academy of Sciences, Pushchino 142290, Russia; nina_uspalenko@mail.ru (N.I.U.); makcvel.95@mail.ru (A.A.M.); nat-niig@yandex.ru (N.V.K.); pavlikl@mail.ru (L.L.P.); nata.imagination@gmail.com (N.V.B.); nkhunderyakova@gmail.com (N.V.K.); shigaeva-marija@rambler.ru (M.I.S.); vasilisa.medv@mail.ru (V.P.M.); malkovae@gmail.com (A.E.M.); vkitchigina@gmail.com (V.F.K.); 2 Pushchino State Natural Science Institute, Pushchino 142290, Russia

**Keywords:** Parkinson’s disease, 6-hydroxydopamine, uridine, mitochondria, mitochondrial ATP-dependent potassium channel, oxidative stress, structure, neurons, synapses

## Abstract

Using a model of Parkinson’s disease (PD) induced by the bilateral injection of neurotoxin 6-hydroxydopamine (6-OHDA) into rat brain substantia nigra (SN), we showed uridine to exert a protective effect associated with activation of the mitochondrial ATP-dependent potassium (mitoK-ATP) channel. Injection of 4 µg neurotoxin evoked a 70% decrease in the time the experimental animal spent on the rod in the RotaRod test, an increase in the amount of lipid peroxides in blood serum and cerebral-cortex mitochondria and the rate of reactive oxygen species formation, and a decrease in Ca^2+^ retention in mitochondria. Herewith, lymphocytes featured an increase in the activity of lactate dehydrogenase, a cytosolic enzyme of glycolysis, without changes in succinate-dehydrogenase activity. Structural changes occurring in the SN and striatum manifested themselves in the destruction of mitochondria, degeneration of neurons and synapses, and stratification of myelin sheaths in them. Subcutaneous injections of 30 µg/kg uridine for 22 days restored the neurotoxin-induced changes in these parameters to levels close to the control. 5-Hydroxydecanoate (5 mg/kg), a specific mitoK-ATP channel inhibitor, eliminated the beneficial effect of uridine for almost all characteristics tested, indicating the involvement of the mitoK-ATP channel in the protective effect of uridine. The mechanism of the protective effect of uridine and its therapeutic applications for the prevention and treatment of PD are discussed.

## 1. Introduction

Parkinson’s disease (PD) is a neurodegenerative disorder the probability of the development of which significantly increases with age. It is expected that, as a result of overall aging of the world population, the number of patients with PD will significantly increase [1]. Elucidation of the mechanisms of its occurrence and search for new approaches to its prevention and treatment are an urgent problem. The most common method of PD treatment to date has been the dopaminergic replacement therapy with levodopa, which only temporarily mitigates the symptoms of motor deficits [2].

One of the main processes underlying neurodegenerative disorders, in particular, PD, is mitochondrial dysfunction. Taking into account the high energy needs of the body, especially of the brain, this condition may have devastating consequences for the survival of neurons [3].

The mitochondrial dysfunction in brain tissues is commonly caused by an excessive production of reactive oxygen species (ROS), whose level increases with age [4]. At the same time, an increase in the ROS content in the substantia nigra (SN) and striatum induces the oxidation of dopamine accompanied by the death of dopaminergic neurons, which is a cause of PD development [5]. The lack of a radical therapy encourages the development of new approaches to the prevention and treatment of PD using currently available experimental models.

Since PD patients are found to feature an inhibition of respiratory chain complex I in mitochondria [5], one of the methods of modeling PD is the use of 6-hydroxydopamine (6-OHDA), which inhibits this complex [5,6]. This neurotoxin is unable to cross the hematoencephalic barrier and is therefore administered directly into the SN or striatum. Entering the dopaminergic neurons with the help of a dopamine transporter, 6-OHDA initiates an increase in the amount of ROS in them [6]. This can damage the protein structures of the brain tissue, affect the function of neurons, and lead to motor impairments [4]. An increase in the level of ROS also results in the oxidation of dopamine followed by its degradation and, as a consequence, severe mitochondrial dysfunction [7].

We have previously found that the injection of uridine to animals leads to an increase in the concentration of uridine diphosphate (UDP), a natural activator of the mitochondrial ATP-dependent potassium (mitoK-ATP) channel, which normalizes oxidative exchange and eliminates mitochondrial dysfunction [8,9,10,11].

In this work, we studied the therapeutic action of uridine in a model of neurotoxin-induced PD in rats and the degree of the involvement of the mitoK-ATP channel in the development of Parkinson’s syndrome.

## 2. Results

### 2.1. Effect of Uridine on the Behavior of Animals in a Model of PD Created by the Bilateral Injection of 6-OHDA into the Substantia Nigra

**Figure 1:**
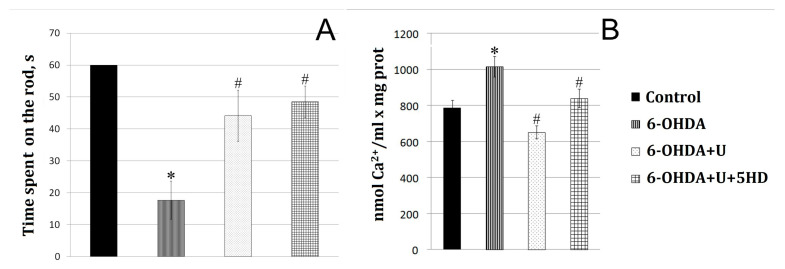
Effect of uridine on the motor function and ion exchange in rat brain mitochondria after the bilateral injection of 6-OHDA into the substantia nigra. (**A**) Estimation of the motor function on the 22nd day after the injection. *y*-axis: the time the animals move at a speed of 8 cm/s before falling from the RotaRod device. (**B**) Calcium retention capacity of rat brain mitochondria in the normal state and in a PD model. The results are presented as the mean ± SEM. * *p* < 0.05, relative to the control, # *p* < 0.05, relative to the 6-OHDA group.

In a model of PD created by the bilateral injection of 6-OHDA into the rat brain SN, we observed a disturbance of the dopaminergic nigrostriatal pathway and an impairment of the motor function. Nissl staining used to quantitatively assess the fate of dopaminergic neurons in the substantia nigra pars compacta after administration of the neurotoxin revealed that 70% of the neurons died. Upon creation of the model, the rats were in a calm environment with free access to water and food; in the postoperative period, they were under daily control. Additionally, the animals were administered Ringer’s solution (5 mL per day). Motor impairments were assessed using the RotaRod behavioral test on the 22nd day after the injection of the neurotoxin (Figure 1A).

Administration of the neurotoxin decreased by 70% the time spent by the animal on the rotating rod compared to the control group (Figure 1A). To correct 6-OHDA-induced impairments, uridine was injected intraperitoneally at a concentration of 30 mg/kg of body weight. After the injection of the neurotoxin together with uridine, the time spent on the rod increased to approach that of the control values. 5-Hydroxydecanoate (5-HD), a specific inhibitor of the mitoK-ATP channel, did not eliminate the protective effect of uridine within the time the animal was on the rotating rod.

### 2.2. Effect of Uridine on the Calcium Exchange in Rat Brain Mitochondria after the Bilateral Administration of 6-OHDA into the Substantia Nigra

Disturbance of calcium homeostasis observed in PD is known to lead to cell death. The cell death is, presumably, associated with the overload of mitochondria with calcium, which promotes the development of oxidative stress in them and changes the permeability of the mitochondrial membrane followed by the opening of a nonspecific pore and the death of dopaminergic neurons [12,13].

The results of the present study showed that the administration of the neurotoxin to model PD decreased the calcium retention capacity of mitochondria by 63% relative to the control. Injection of uridine at a concentration of 30 mg/kg of body weight together with 6-OHDA increased the calcium retention capacity of mitochondria to the control level. Application of 5-HD completely eliminated the beneficial action of uridine (Figure 1B).

### 2.3. Effect of Uridine on the Oxidative Exchange in Rat Brain Mitochondria after the Bilateral Injection of 6-OHDA into the Substantia Nigra

**Figure 2:**
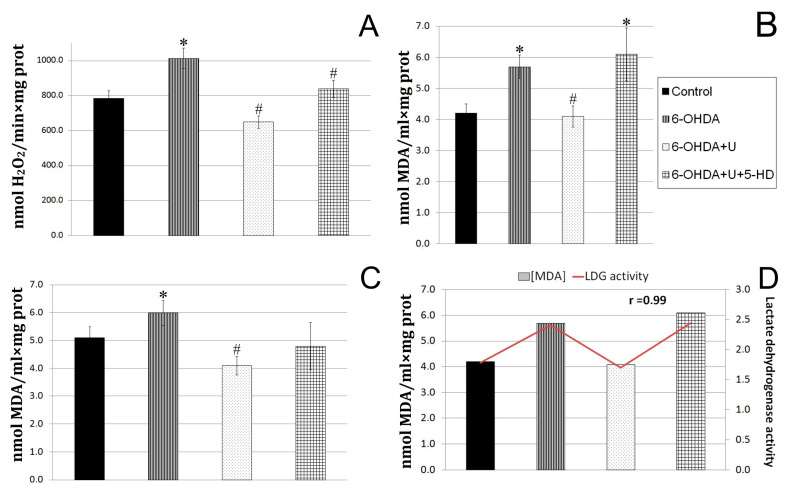
Effect of uridine on the rate of hydrogen peroxide formation and MDA production in rat brain mitochondria and blood serum after the bilateral injection of 6-OHDA into the substantia nigra. (**A**) The rate of hydrogen peroxide formation by rat brain mitochondria on the 22nd day after the administration of 6-OHDA. (**B**) Concentration of lipid peroxidation products in rat brain mitochondria on the 22nd day after the injection of 6-OHDA. (**C**) Concentration of lipid peroxidation products in blood serum of rats on the 22nd day after the injection of 6-OHDA. (**D**) A correlation between the content of TBA-active products in the blood serum and the LDG activity in lymphocytes. The results are presented as the mean ± SEM. * *p* < 0.05, relative to the control, # *p* < 0.05, relative to the 6-OHDA group.

The rate of hydrogen peroxide formation in the experiments was determined in a system with Amplex Red and horseradish peroxidase. It was found that the neurotoxin increased the rate of hydrogen peroxide formation in rat brain mitochondria in the experimental group by 23% compared to the control groups. The injection of uridine together with the neurotoxin decreased the characteristic to values even below the control, and the injection of 5-HD in combination with uridine eliminated the protective effect of uridine, increasing the rate of hydrogen peroxide formation (Figure 2A).

When diffusing through biological membranes, hydrogen peroxide can react with unsaturated fatty acids of lipids, which leads to the destruction of membranes. One of the products of these reactions is malondialdehyde (MDA). In this work, we determined the content of lipid peroxidation products in brain mitochondria (Figure 2B). In rats injected with the neurotoxin, the content of MDA was, by 18%, higher than in the control group. The injection of uridine decreased the MDA content in brain mitochondria to a level significantly lower than in the control (Figure 2B), which is consistent with our data indicating a decrease in the rate of hydrogen peroxide formation in the brain (Figure 2A).

The measurements showed that the MDA content in the blood serum of rats with PD increased by 37%, and the treatment with uridine lowered this characteristic to the control level (Figure 2C). At the same time, the injection of 5-HD to animals receiving uridine treatment eliminated the effect of uridine in the blood serum and increased the MDA level by 45% relative to the MDA content in the group of animals treated with uridine.

### 2.4. Effect of Uridine on Succinate Dehydrogenase and Lactate Dehydrogenase Activities in Blood Lymphocytes in a PD Model

A cytobiochemical determination of the activity of the key mitochondrial enzyme, succinate dehydrogenase (SDH), in immobilized blood lymphocytes in a blood smear from PD rats did not reveal any significant differences from the control. After the injection of uridine to sick animals, the activity of SDH increased by 80% compared to the control. The application of 5-HD reduced the beneficial effect of uridine. Measurements of the activity of the glycolysis enzyme, lactate dehydrogenase (LDH), in blood lymphocytes in a smear from PD rats showed that it increased by 35% compared to the control, and the treatment with uridine decreased the activation to that of the control value. The administration of the 5-HD inhibitor abolished the effect of uridine, increasing the LDH level by 37% to the level in the group of sick rats. Thus, in the mitochondria of blood lymphocytes from rats with PD, glycolysis dominated over oxidative phosphorylation; the administration of uridine enhanced mitochondrial respiration by 80% and reduced glycolysis. The injection of 5-HD completely abolished the effect of uridine (Table 1).

The characteristics of glycolysis (activity of LDH) in lymphocytes and oxidative stress (MDA level) in serum were revealed to have a direct positive correlation with coefficient *r* = 0.99. In animals with Parkinson’s syndrome, the LDH activity increased by 37%, and the MDA content rose by 35%. Treatment with uridine decreased both characteristics to the values for the control group, and 5-HD completely abolished the effect of uridine in lymphocytes and serum. The reduction in SDH activity and the increase in LDH indicate the disturbance of energetic processes in lymphocytes in PD, which shows up in the retardation of aerobic oxidation and enhancement of glycolysis; this is in line with the literature data [14].

### 2.5. Ultrastructures of Substantia Nigra and Striatum Neurons after the Bilateral Administration of 6-OHDA and the Protective Effect of Uridine on These Structures

**Figure 3:**
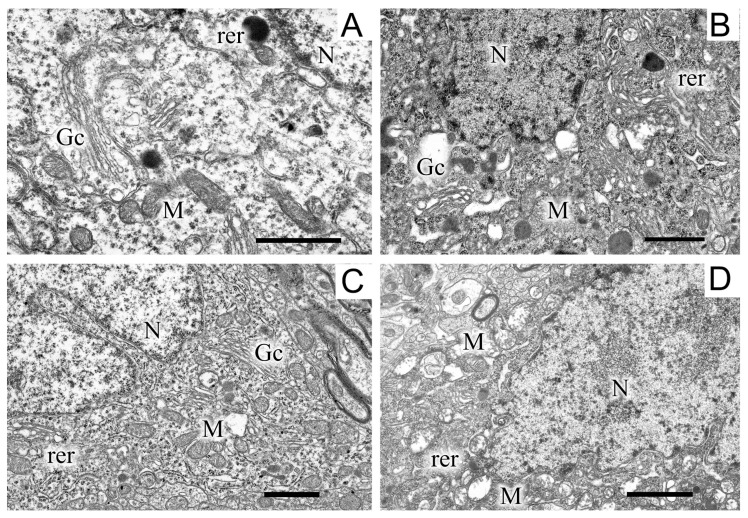
A region of a dopaminergic neuron from the substantia nigra in the control (**A**), in a model of parkinsonism (**B**), in the presence of uridine (**C**), and in the presence of uridine and 5-HD (**D**). N, nucleus; Gc, Golgi complex; M, mitochondrion; rer, endoplasmic reticulum. Scale bar = 1 µm.

Images of the ultrastructure of the dopaminergic neuron in the SN were obtained 22 days after the induction of Parkinson’s syndrome in rats by the bilateral injection of 6-OHDA into the SN (Figure 3).

Exposure to the neurotoxin resulted in the appearance of a large number of dark neurons (Figure 3B). As compared with the control (Figure 3A), where the neurons of the SN had a light cytoplasm and a light nucleus, the neurons from experimental animals had a high electron density of the cytoplasm, a dense nucleus with a convoluted sheath, enlarged cisterns of both the endoplasmic reticulum and the Golgi complex, as well as a disordered structure of mitochondria in which vacuoles appeared. The cytoplasm of a neuron contained a large number of lysosomes and lipofuscin granules. The number of mitochondria significantly (by almost 1.5 times) increased as compared with the control (2.22 ± 0.18 in the experiment, 1.13 ± 0.09 in the control). Administration of uridine prevented the structure of neurons from becoming damaged; as a result, they had a morphology similar to that of neurons in the control group (Figure 3C). They had a light cytoplasm and a light nucleus with flattened cisternae of the reticulum. The structure of their mitochondria was not damaged. Administration of the mitoK-ATP channel inhibitor, 5-HD, together with uridine abolished the beneficial effect of uridine: neurons with a dense cytoplasm and with mitochondria having abnormalities in the vicinity of cristae appeared, vacuoles emerged, and the lysis of the stroma occurred (Figure 3D). Uridine also decreased the number of mitochondria in presynaptic endings to that of the control value (1.2 ± 0.08), and 5-HD eliminated the effect of uridine, decreasing their number to the level recorded in pathology (2.3 ± 0.2).

**Figure 4:**
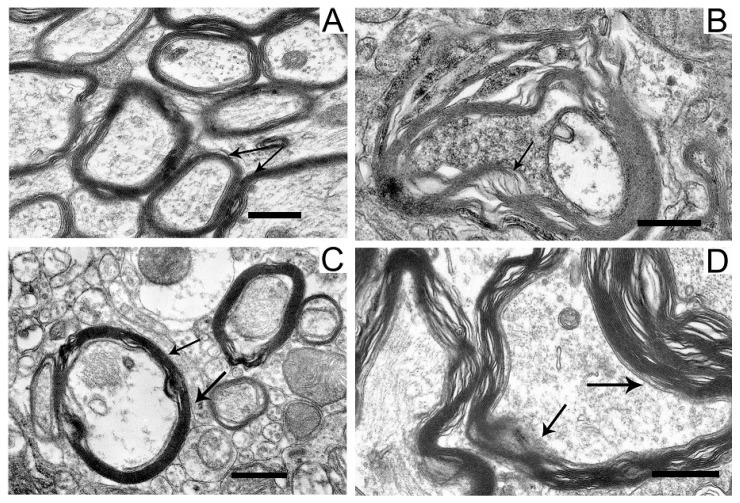
Myelin sheaths of axons in the neuropil of substantia nigra neurons in the control (**A**), in a model of parkinsonism (**B**), in the presence of uridine (**C**), and in the presence of uridine and 5-HD (**D**). Myelin sheaths are shown by arrows. Scale bar = 0.5 µm.

In the neuropil of SN neurons, we observed significant structural damage to the myelin sheaths of axons (Figure 4B,D).

Compared with the control (Figure 4A), in neurons damaged by the neurotoxin, we noted a pronounced stratification of membrane layers of the myelin sheath and the protrusion of its part toward the cytoplasm (Figure 4B). After the administration of uridine, the myelin structure remained similar to that in the control (Figure 4C). Administration of 5-HD together with uridine abolished the protective effect of uridine; in this case, the myelin sheaths of axons remained untwisted (Figure 4D).

**Figure 5:**
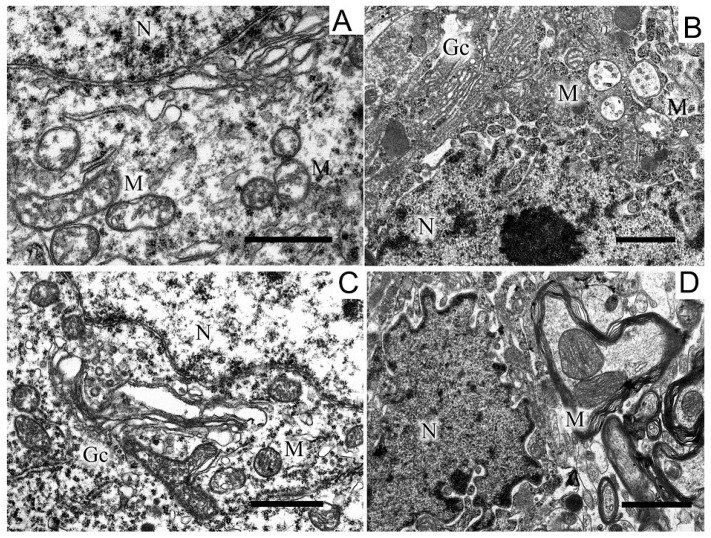
A region of a neuron from the striatum in the control (**A**), in a model of parkinsonism (**B**), in the presence of uridine (**C**), in the presence of uridine and 5-HD (**D**). N, nucleus; Gc, Golgi complex; M, mitochondrion; rer, endoplasmic reticulum. Scale bar = 1 µm.

Along with disruptions in the SN structure, we observed changes in the striatum. They were in many respects similar to those found in the SN and consisted in the appearance of dark neurons with a dark cytoplasm and a dark nucleus, which had an untwisted sheath (Figure 5).

The cisternae of the reticulum and the Golgi complex in the cytoplasm were dilated (Figure 5B) as compared with the light cytoplasm and the light nucleus in the control (Figure 5A). After treatment with uridine, the structure of a neuron became close to that of the intact neuron, with a light cytoplasm and a light nucleus and flattened cisternae of the Golgi complex and the reticulum (Figure 5B). 5-HD abolished the protective effect of uridine (cf. Figure 5B,D). In the 6-OHDA group of animals, in the neurons of the striatum, we observed an increase in the number of mitochondria in presynaptic endings by 1.5 times compared with the control (2.7 ± 0.2). After the administration of uridine, their number remained at the control level (1.7 ± 0.1), and in the group of animals treated with uridine in combination with 5-HD, it did not differ from the value for experimental animals (2.2 ± 0.2).

**Figure 6:**
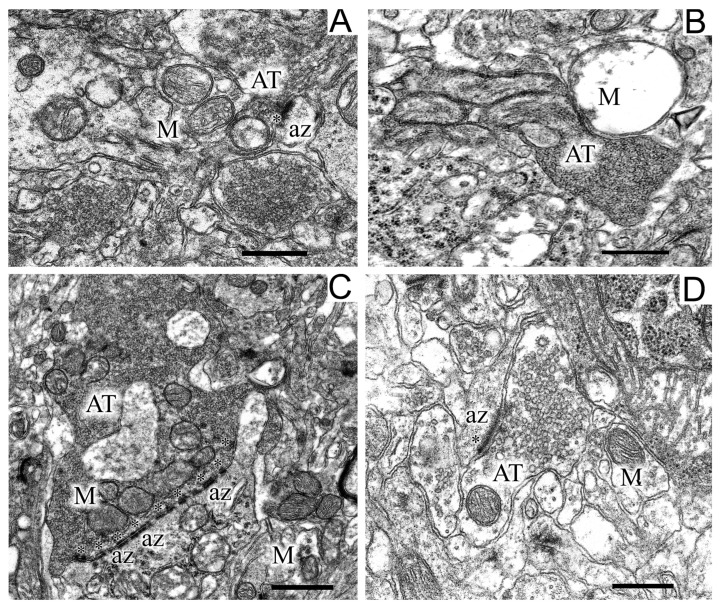
Regions of the neuropil of striatum neurons in the control (**A**), in a model of parkinsonism (**B**,**C**), and after the injection of uridine (**D**). Panel (**B**) shows a destroyed, swollen mitochondria and a large dark axon terminus, panel (**C**) shows synaptic endings with perforated multiple postsynaptic densities. AT, axon terminus; M, mitochondrion; az, active zone (asterisks). Scale bar = 1 µm.

The termini of axons of dopaminergic neurons of the SN commonly form axo-dendritic synapses in the striatum. Analysis of the state of axons in the striatum in control (Figure 6A) and experimental (Figure 6B,C) animals showed that the modeling of parkinsonism induced a significant restructuring of axo-dendritic synapses in this area.

In experimental rats, dilated axo-dendritic synaptic endings with significant accumulation of synaptic vesicles were found (Figure 6C). Synaptic vesicles in these synapses were packed very densely to form a continuous conglomerate. Along with these changes, the degeneration of axons by the “dark type” was observed in some places in the striatum of experimental animals (Figure 6B). As seen in Figure 6B, degenerating axons had a high electron density and swollen mitochondria. In the striatum of experimental animals, synaptic endings with perforated multiple postsynaptic indurations were found (Figure 6B). It should be noted that, in control animals, only single postsynaptic indurations were encountered in postsynaptic endings (Figure 6A). Uridine prevented the dilatation of axo-dendritic synaptic endings and the accumulation of synaptic vesicles in them, the degradation of axons, the swelling of mitochondria localized therein, and the appearance of multiple perforated postsynaptic indurations (Figure 6C).

**Figure 7:**
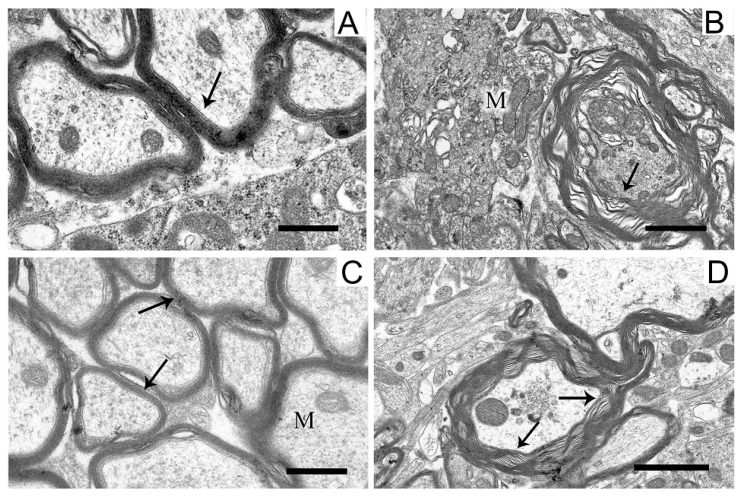
Myelin sheaths of axons in the neuropil of the striatum in the control (**A**), in a model of parkinsonism (**B**), in the presence of uridine (**C**), and in the presence of uridine and 5-HD (**D**). Myelin sheaths are shown by arrows. M, mitochondrion. Scale bar = 0.5 µm.

The structure of myelin sheaths in the neuropil of striatal neurons was also disordered in the rat PD model (Figure 7).

Sheaths with stratified lamellae and membrane protrusions appeared (Figure 7B). After the administration of uridine (Figure 7C), the structure of myelin remained resistant to damage and retained a close-to-intact appearance (for Figure 7A). The injection of 5-HD eliminated the protective effect of uridine (Figure 7D), and stratified myelin lamellae were seen.

## 3. Discussion

The work proposes a new approach to the prevention and treatment of PD. The approach is based on the activation of the mitoK-ATP channel by its natural activator, which leads to an enhancement of energy metabolism, normalization of oxidative metabolism in brain tissues, and, as a consequence, a decrease in mitochondrial dysfunction developing in this disease.

A characteristic sign of PD is the progressive degeneration of dopaminergic neurons in the compact part of the SN and striatum, which leads to a decline in the motor activity of animals [4,7]. Uridine administered after the injection of neurotoxin 6-OHDA was found to increase the motor activity of rats more than threefold as compared with that for the 6-OHDA group (Figure 1A). This may presumably be due to the known ability of uridine to increase the synthesis of coenzyme A (CoA) and thereby to activate the functioning of the Krebs cycle, i.e., to enhance the energy metabolism in the rat body [15]. It also follows from the data obtained that the calcium metabolism in brain mitochondria of experimental animals is disturbed, which may contribute to the opening of cyclosporin-sensitive pores in them. In turn, the opening of the pore leads to cell death by necrosis or apoptosis [12].

PD is also characterized by an oxidative metabolism disturbance in brain tissues expressed in the accumulation of ROS, as well as by an increase in the rate of hydrogen peroxide production. This is probably due to the fact that, being structurally similar to dopamine, 6-OHDA enters the cell by means of dopamine and norepinephrine transporters, where it increases the concentration of ROS both enzymatically (using mitochondrial monoamine oxidase B) and nonenzymatically [4]. Furthermore, as rotenone, it is capable of inhibiting the function of the respiratory chain complex I, which also leads to the accumulation of peroxide radicals. An increase in the amount of ROS in tissues causes the oxidation of intracellular-compartment structures of dopaminergic neurons, as well as the oxidation of dopamine, which is manifested in the appearance of cognitive and motor impairments typical of PD [1,4,16].

We used uridine to correct biochemical impairments caused by the administration of 6-OHDA. At the concentrations applied, this nucleoside prevents the development of hypoxia in tissues, as shown in our previous research [11]. Administration of uridine to rats with Parkinson’s syndrome completely prevented ion exchange disorders in these organelles, as well as a neurotoxin-induced increase in the formation rate and number of peroxide compounds in mitochondria and blood serum. At the same time, the energy metabolism in lymphocytes was also normalized.

Modeling of PD in rats revealed significant changes in the structures of rat brain SN and striatum. Each of these regions is of critical significance for the development of PD. Their structures are part of the extrapyramidal motor system, which is responsible for the regulation of motor function and muscle tone and is involved in many vegetative functions, such as respiration, cardiac activity, and blood vessel tone [17].

Morphological changes are observed in neurons, presynaptic endings, and in the myelin sheath of the SN and striatum. These changes may be a cause of a decrease in energy efficiency and ATP level in brain tissues, which leads to progressive degeneration of dopaminergic neurons in the compact region of the SN and striatum [18]. An increase in the number of mitochondria in synaptic endings may be an indicator of an enhancement of their activity aimed at maintaining the mechanisms of plasticity. It is assumed that the dysfunction of mitochondria and an increase in their number followed by the induction of apoptosis markers may be associated with the accumulation of mitochondrial DNA mutations due to the impact of ROS [19].

It is considered that neurodegeneration begins in synaptic junctions, as they are the first to be damaged in PD [20]. An increase in the number of perforated synapses in the PD model found in this work is usually considered to be a compensation for the degeneration of dopaminergic cells, which leads to a delay in or prevention of the development of the disease [21]. Similar plastic changes in synaptic endings of the striatum have been previously observed in a model of induced autoimmune synucleinopathy [22].

Upon administration of the neurotoxin, we observed a disruption of myelin sheaths known to be required for the separation of nerves from one another and the enhancement of nerve pulse transmission. At these structural disorders, signals become mixed, and the normal movements of the animal become impossible [23]. The above-described disorders in SN and striatum neurons, as well as in myelin sheaths, have been also found in our earlier work on a model of PD induced by rotenone, an inhibitor of the respiratory chain complex I [10], which indirectly indicates the identity of the pathology created in these models.

The study using the inhibitory analysis shows that the mechanism by which uridine affects the biochemical parameters and structure of experimental animals’ brains is associated to a greater extent with the activation of the mitoK-ATP channel, because 5-HD, the channel inhibitor, abolishes the positive effect of uridine almost completely. We explain this by an increase in the concentration of UDP, an activator of this channel, in tissues at the administration of uridine [24]. It is known that UDP does not enter the cell but forms in tissues from uridine, which penetrates through the hematoencephalic barrier [8,9]. Activation of the channel is known to enhance the cyclization of potassium in mitochondria, which lowers their membrane potential (ΔΨ_m_) [25,26]. It has been shown earlier that a 10% decrease in ∆Ψ_m_ diminishes the rate of peroxide formation in mitochondria sevenfold [27].

As the effect of uridine on brain tissues is almost completely eliminated by 5-HD, a selective inhibitor of the mitoK-ATP channel, we suggest that this channel is involved in maintaining metabolic processes and integrity of the brain structure in PD and, probably, in the development of the disease. The assumption about the role of the channel in the development of PD is consistent with our earlier data on a significant (3–5-fold) decrease in the activity of this channel with age [28]. The fact that the incidence of PD is generally recognized to rise with age [1,29] confirms the existence of a correlation between the disturbance of the mitoK-ATP channel function and the development of PD. Thus, the data obtained in the present research are indicative of the involvement of mitochondria and the mitoK-ATP channel in the development of PD.

Still, as 5-HD did not always completely abolish the effect of uridine, other metabolic pathways of this nucleoside can be also assumed to be involved in protecting the rat body from the neurotoxin. One of such pathways, as we noted above, can be the synthesis of acetyl-CoA from uridine, which is important for the recovery of the Krebs cycle energy equivalents, NADH and FADN_2_, known to be depleted during the development of oxidative stress in toxic models of PD [15]. In this regard, the administration of uridine can significantly enhance the energy and ion metabolism in mitochondria, which prevents motor dysfunction in sick animals. The mechanism of the protective action of uridine in PD can also be related to the formation of UDP glucose involved in glycogen synthesis [30,31]. As for the effect of uridine on the prevention of structural changes in myelin in our PD model, this can be associated with an increased formation of cytidine triphosphate (CTP) from UTP, the concentration of which increases after the administration of uridine [8,9]. An increase in CTP in tissues stimulates the synthesis of brain phospholipids, which, probably, does prevent the breakdown of the myelin sheath [32].

Since the mitoK-ATP channel inhibitor eliminated the beneficial effect of uridine in almost all cases, we assume that the main mechanism of action of this metabolite is through the activation of the channel. The activation of the channel leads to an acceleration of potassium cyclization in mitochondria, which lowers their membrane potential to induce a “mild uncoupling”. Herewith, the rate of peroxide formation in mitochondria decreases, oxidative metabolism normalizes, and both the calcium overload of cells and the neurotoxin-induced structural changes in the brain tissue are prevented.

## 4. Materials and Methods

Wistar male rats (N = 23) weighing 190–220 g were used. The animals were housed in a vivarium at the Institute of Theoretical and Experimental Biophysics, Russian Academy of Sciences. All manipulations with animals were carried out in compliance with the Council Directive 2010/63 EU of the European Parliament and the Regulations of the European Convention for the Protection of Vertebrate Animals Used for Scientific Purposes (Strasbourg, France).

### 4.1. Stereotaxic Intervention and Groups of Animals

In this series of experiments, animals were divided into four groups: (1) the control group; to the SN of both brain hemispheres, 4 µL of a 0.2% L-ascorbic acid solution was injected (N = 5); (2) the 6-OHDA group; 4 µg of neurotoxin 6-OHDA dissolved in 6 µL of a 0.2% L-ascorbic acid solution was injected (N = 6); (3) the 6-OHDA + U group; after the injection of 6-OHDA, the mitoK-ATP channel activator, uridine (U), was daily administered intraperitoneally for 22 days at a dose of 30 mg/kg of body weight (N = 6); (4) the 6-OHDA+U + 5-HD group; after the injection of 6-OHDA, uridine (U) was administered daily, and 15 min after the injection of uridine, the mitoK-ATP channel inhibitor, 5-HD, was subcutaneously injected at a dose of 5 mg/kg of body weight (N = 6).

For anesthesia, Xylozin (4 mg/kg of body weight) and Zoletil (60 mg/kg) were used. 6-OHDA (5 mg) was dissolved in 0.8 mL of 300 mM sodium chloride (a sterile saline infusion solution) with 0.1% ascorbic acid. Operations were carried out on a stereotaxic frame using a 10 mL micro syringe (Hamilton Co., Reno, NV, USA) by the method described by Torres and Dunnett [33]. The neurotoxin was injected bilaterally along the coordinates (anterior-posterior  =  −2.0 mm, lateral  = −5.2 mm, ventral = −8.2 mm) [34].

### 4.2. Behavioral Testing

The endurance and motor coordination of animals were assessed on a RotaRod device (Neurobotics, Moscow, Zelenograd, Russia) on the 22nd day after surgery. The facility was a rotating horizontal rod with lateral vertical baffles. An experimenter placed a rat into the facility and controlled the running of the animal. The time during which the animal stayed on the rotating rod (speed 8 cm/s) was measured.

### 4.3. Isolation of Mitochondria

Rats were decapitated on the 22nd day after surgery. Brain tissue fragments were washed and homogenized by a manual homogenizer in an isolation medium (230 mM mannitol, 70 mM sucrose, 1.0 mM EDTA, and 10 mM HEPES-KOH, pH 7.4). Homogenates were centrifuged at 12,000× *g* for 10 min. The supernatant was poured into a new test tube and centrifuged again at 12,000× *g* for 10 min. Then, the supernatant was removed and the sediment was homogenized to a homogenous state in a washing medium (0.075 M sucrose, 0.225 M mannitol, 10 mM HEPES-KOH, pH 7.4) and centrifuged at 12,000× *g* for 10 min. The resulting sediment was collected and further homogenized with the washing medium. All procedures were carried out on ice in a cold room at +4 °C.

### 4.4. Calcium Retention Capacity

The Ca^2+^ retention capacity of mitochondria from the brain of rats in the norm and in experimental parkinsonism was determined using a Record-4 potentiometric system with a Ca^2+^-selective electrode (NikoAnalit, Moscow, Russia). The incubation medium contained 150 mM KCl, 1 mM NaH_2_PO_4_, 10 µM EGTA, and 10 mM HEPES-KOH (pH 7.4). The concentration of the mitochondrial protein in a cuvette was 1 mg/mL. The substrates used were 5 mM malate and 5 mM potassium gluconate. CaCl_2_ was added in fractions of 25 µM every 30 s. The calcium retention capacity was calculated as nmol Ca^2+^/mL × mg protein.

### 4.5. Rate of H_2_O_2_ Formation in Brain Mitochondria

The generation of hydrogen peroxide by rat brain mitochondria was measured using a fluorimeter (Varian Medical Systems, Palo Alto, CA, USA) at an excitation of 570 nm and an emission of 585 nm. The reaction mixture contained 120 mM KCl, 5 mM KH_2_PO_4_, 10 mM HEPES-KOH, and 0.5 mM EGTA, pH 7.4. The hydrogen peroxide production was measured at 37 °C in the presence of 20 µM Amplex Red and 2 U/mL horseradish peroxidase. To avoid the impact of optical effects associated with mitochondrial swelling, the activity of mitochondria was measured using the two-wave mode of registration (572–600 nm) [35]. The concentration of mitochondria in the measurement medium was 0.2 mg/mL. The rate was expressed in nmol H_2_O_2_/min × mg protein.

### 4.6. Concentration of Lipid Peroxidation Products in Brain Mitochondria and Blood Serum

The concentration of lipid peroxidation products in rat brain mitochondria and blood serum was determined from the reaction of 250–275 µL of 5% SDS, 900 µL of 20% CH_3_COONa, 300 µL of a 0.8% solution of thiobarbituric acid (TBA), and 50–25 µL serum or mitochondria. Eppendorf dishes with the reaction mixture were incubated for 1 h at 90 °C. Then, the liquid was centrifuged at 2000× *g* for 5 min. The product was a trimethine complex of characteristic pink color (λ_max_ = 532 nm). The measurement was performed on a spectrophotometer (Shimadzu Europa GmbH, Kyoto, Japan) at 532–650 nm. The results were expressed in nmol/min × mg protein.

### 4.7. Succinate Dehydrogenase and Lactate Dehydrogenase Activities in Lymphocytes

The activities of succinate dehydrogenase (SDH) in mitochondria in peripheral blood and lactate dehydrogenase (LDH) in the cytosol in lymphocytes in peripheral blood were determined by a biocytochemical method (license no. 2364868, Russia) from the reduction in nitro blue tetrazolium (NBT) to dark blue formazan [36]. Smears were prepared on a Blood Smear Preparation Device (Microscopy Vision, Dornbirn, Austria), dried for 30–60 min, fixed in a 60% solution of acetone buffered with 10 mM HEPES (Sigma, St. Louis, MO, USA) (pH 5.2–5.5) for 30 s, rinsed with distilled water, and dried. Then, smears were incubated at 37 °C for 1 h in a medium containing 125 mM KC1, 10 mM HEPES, and 1 mg/mL of NBT, pH 7.2 ± 0.05. The incubation medium was supplemented with 5 mM lactic acid, 5 mM malonic acid, and 0.5 mM NADH to determine the activity of LDH and with 5 mM succinic acid to determine the activity of SDH. Smears were washed with distilled water, dried, and stained in a 0.05% neutral red solution. Samples were examined at a magnification of 10 × 100. On each smear, 100 cells collected from the edge of the smear were examined. Samples were photographed using the program BioImages, which makes it possible to measure the mean area of a smear stained by diformazan (in µm^2^) in a sample of 100 lymphocytes.

### 4.8. Transmission Electron Microscopy

After decapitation, pieces of the SN were taken from rats. The material was fixed immediately, by placing tissue samples in a 2.5% solution of glutaraldehyde in 0.1 M cacodylate buffer (pH 7.4). Postfixation was performed with 2% osmium tetroxide in 0.1 M cacodylate buffer (pH 7.4). Dehydration was carried out in alcohols of increasing concentration and acetone. Dehydrated samples were embedded in Epon 812 resin. Ultrathin sections 60–70 nm thick were made on an ultramicrotome (Leica EM UC7, Wetzlar, Germany). Sections were contrasted with 1% uranyl acetate and zinc citrate. Samples were scanned using a transmission electron microscope (JEOL JEM-100B, Tokyo, Japan). The total number of mitochondria (nMch) on microphotographs was calculated.

### 4.9. Statistical Analysis

All values are given as the means ± standard error of the means. Statistical analysis was performed by the paired *t*-test. Differences are significant at * *p* < 0.05 and ** *p* < 0.01. MS Excel, ImageJ (Java 1.6.0_12, RRID: SCR_003070, LOCI, University of Wisconsin, Madison, Wisconsin, USA), Origin 2016 (OriginLab, Northampton, MA, USA), and Prism GraphPad 7 (GraphPad Software, RRID: SCR_002798) softwares were used for data and statistical analysis.

## 5. Conclusions

Based on the data obtained, it was concluded that the natural metabolite uridine protects the rat body against impairments induced by the administration of neurotoxin 6-OHDA and prevents negative consequences observed when modeling PD. The mechanism of the protective effect of uridine is mainly associated with the activation of the mitoK-ATP channel, which reduces the oxidative stress and the mitochondrial dysfunction in tissues. In this connection, uridine can be considered to be a metabolite that prevents the development of PD and can be used as a therapeutic means at its early stages.

## Figures and Tables

**Table 1:** Succinate dehydrogenase and lactate dehydrogenase activities in lymphocytes in experimental parkinsonism.

Group	SDH Activity, Relative Units	LDH Activity, Relative Units
Control	1.14	1.78
6-OHDA	1.37	2.41 *
6-OHDA+U	1.82 *	1.70
6-OHDA+U + 5-HD	1.00	2.44 *

* *p* < 0.05, relative to the control.

## Data Availability

The data presented in this study are available on request from the corresponding author.

## References

[1] Balestrino R., Schapira A.H.V. (2020). Parkinson disease. Eur. J. Neurol..

[2] Lee T.K., Yankee E.L. (2021). A review on Parkinson’s disease treatment. Neuroimmunol. Neuroinflamm..

[3] Rose J., Brian C., Woods J., Pappa A., Panayiotidis M.I., Powers R., Franco R. (2017). Mitochondrial dysfunction in glial cells: Implications for neuronal homeostasis and survival. Toxicology.

[4] Anik M.I., Mahmud N., Masud A.A., Khan M.I., Islam M.N., Uddin S., Hossain M.K. (2022). Role of reactive oxygen species in aging and age-related diseases: A review. ACS Appl. Bio Mater..

[5] Gao X.Y., Yang T., Gu Y., Sun X.H. (2022). Mitochondrial dysfunction in Parkinson’s disease: From mechanistic insights to therapy. Front. Aging Neurosci..

[6] Barata-Antunes S., Teixeira F.G., Mendes-Pinheiro B., Domingues A.V., Vilaça-Faria H., Marote A., Silva D., Sousa R.A., Salgado A.J. (2020). Impact of aging on the 6-OHDA-induced rat model of Parkinson’s disease. Int. J. Mol. Sci..

[7] Burbulla L.F., Song P., Mazzulli J.R., Zampese E., Wong Y.C., Jeon S., Santos D.P., Blanz J., Obermaier C.D., Strojny C. (2017). Dopamine oxidation mediates mitochondrial and lysosomal dysfunction in Parkinson’s disease. Science.

[8] Krylova I.B., Selina E.N., Bulion V.V., Rodionova O.M., Evdokimova N.R., Belosludtseva N.V., Shigaeva M.I., Mironova G.D. (2021). Uridine treatment prevents myocardial injury in rat models of acute ischemia and ischemia/reperfusion by activating the mitochondrial ATP-dependent potassium channel. Sci. Rep..

[9] Mironova G.D., Khrenov M.O., Talanov E.Y., Glushkova O.V., Parfenyuk S.B., Novoselova T.V., Lunin S.M., Belosludtseva N.V., Novoselova E.G., Lemasters J.J. (2018). The role of mitochondrial KATP channel in anti-inflammatory effects of uridine in endotoxemic mice. Arch. Biochem. Biophys..

[10] Mosentsov A.A., Rozova E.V., Belosludtseva N.V., Mankovskaya I.N., Putiy Y.V., Karaban I.N., Mikheeva I.B., Mironova G.D. (2021). Does the operation of mitochondrial ATP-dependent potassium channels affect the structural component of mitochondrial and endothelial dysfunctions in experimental parkinsonism?. Bull. Exp. Biol. Med..

[11] Krylova I.B., Safonova A.F., Evdokimova N.R. (2018). Correction of hypoxic state by metabolic precursors of endogenous activator of mitochondrial ATP-dependent K^+^ channels. Rev. Clin. Pharmacol. Drug Ther..

[12] Belosludtsev K.N., Dubinin M.V., Belosludtseva N.V., Mironova G.D. (2019). Mitochondrial Ca^2+^ transport: Mechanisms, molecular structures, and role in cells. Biochem. (Mosc.).

[13] Ludtmann M.H.R., Abramov A.Y. (2018). Mitochondrial calcium imbalance in Parkinson’s disease. Neurosci. Lett..

[14] Werner C., Doenst T., Schwarzer M., Schwarzer M., Doenst T. (2015). Metabolic Pathways and Cycles. The Scientist’s Guide to Cardiac Metabolism.

[15] Zhu D., Wei Y., Yin J., Liu D., Ang E.L., Zhao H., Zhang Y. (2020). A pathway for degradation of uracil to acetyl coenzyme A in *Bacillus megaterium*. Appl. Environ. Microbiol..

[16] Silva T.P., Poli A., Hara D.B., Takahashi R.N. (2016). Time course study of microglial and behavioral alterations induced by 6-hydroxydopamine in rats. Neurosci. Lett..

[17] Lu Y., Zhang X., Zhao L., Yang C., Pan L., Li C., Liu K., Bai G., Gao H., Yan Z. (2018). Metabolic disturbances in the striatum and substantia nigra in the onset and progression of MPTP-induced parkinsonism model. Front. Neurosci..

[18] Guo J.D., Zhao X., Li Y., Li G.R., Liu X.L. (2018). Damage to dopaminergic neurons by oxidative stress in Parkinson’s disease (Review). Int. J. Mol. Med..

[19] Todorova V., Blokland A. (2017). Mitochondria and synaptic plasticity in the mature and aging mervous system. Curr. Neuropharmacol..

[20] Soukup S.F., Vanhauwaert R., Verstreken P. (2018). Parkinson’s disease: Convergence on synaptic homeostasis. EMBO J..

[21] Merino-Galán L., Jimenez-Urbieta H., Zamarbide M., Rodríguez-Chinchilla T., Belloso-Iguerategui A., Santamaria E., Fernández-Irigoyen J., Aiastui A., Doudnikoff E., Bézard E. (2022). Striatal synaptic bioenergetic and autophagic decline in premotor experimental parkinsonism. Brain.

[22] Sergeeva T.N., Sergeev V.G., Vezheeva O.A. (2011). Electron-microscopy studies of rat brain neurodegenerative changes in the model of autoimmune synucleopathie. Bull. Udmurt. Univ. Ser. Biology. Earth Sci..

[23] Papuć E., Rejdak K. (2020). The role of myelin damage in Alzheimer’s disease pathology. Arch. Med. Sci..

[24] Mironova G.D., Negoda A.E., Marinov B.S., Paucek P., Costa A.D., Grigoriev S.M., Skarga Y.Y., Garlid K.D. (2004). Functional distinctions between the mitochondrial ATP-dependent K^+^ channel (mitoKATP) and its inward rectifier subunit (mitoKIR). J. Biol. Chem..

[25] Schönfeld P., Gerke S., Bohnensack R., Wojtczak L. (2003). Stimulation of potassium cycling in mitochondria by long-chain fatty acids. Biochim. Biophys. Acta (BBA) Bioenerg..

[26] Garlid K.D., Paucek P. (2003). Mitochondrial potassium transport: The K^+^ cycle. Biochim. Biophys. Acta (BBA) Bioenerg..

[27] Korshunov S.S., Skulachev V.P., Starkov A.A. (1997). High protonic potential actuates a mechanism of production of reactive oxygen species in mitochondria. FEBS Lett..

[28] Shigaeva M., Gritsenko E., Murzaeva S., Gorbacheva O., Talanov E., Mironova G. (2010). Age-related changes in the functioning of the mitochondrial potassium-transporting system. Biofizika.

[29] Strickland M., Yacoubi-Loueslati B., Bouhaouala-Zahar B., Pender S.L.F., Larbi A. (2019). Relationships between ion channels, mitochondrial functions and inflammation in human aging. Front. Physiol..

[30] Belosludtseva N.V., Starinets V.S., Mikheeva I.B., Belosludtsev M.N., Dubinin M.V., Mironova G.D., Belosludtsev K.N. (2022). Effect of chronic treatment with uridine on cardiac mitochondrial dysfunction in the C57BL/6 mouse model of high-fat diet streptozotocin-induced diabetes. Int. J. Mol. Sci..

[31] Skinner O.S., Blanco-Fernández J., Goodman R.P., Kawakami A., Shen H., Kemény L.V., Joesch-Cohen L., Rees M.G., Roth J.A., Fisher D.E. (2023). Salvage of ribose from uridine or RNA supports glycolysis in nutrient-limited conditions. Nat. Metab..

[32] Tracey T.J., Steyn F.J., Wolvetang E.J., Ngo S.T. (2018). Neuronal lipid metabolism: Multiple pathways driving functional outcomes in health and disease. Front. Mol. Neurosci..

[33] Torres E., Dunnett S. (2011). 6-OHDA lesion models of Parkinson’s disease in the rat. Animal Models of Movement Disorders.

[34] Paxinos G., Watson C. (2007). The Rat Brain in Stereotaxic Coordinates.

[35] Ferranti R., da Silva M.M., Kowaltowski A.J. (2003). Mitochondrial ATP-sensitive K^+^ channel opening decreases reactive oxygen species generation. FEBS Lett..

[36] Kondrashova M., Zakharchenko M., Khunderyakova N. (2009). Preservation of the in vivo state of mitochondrial network for ex vivo physiological study of mitochondria. Int. J. Biochem. Cell Biol..

